# Predator-Pest Dynamics of Arthropods Residing in Louisiana Soybean Agroecosystems

**DOI:** 10.3390/insects13020154

**Published:** 2022-01-31

**Authors:** Scott T. Lee, Chaoyang Li, Jeffrey A. Davis

**Affiliations:** 1 Department of Entomology, Louisiana State University Agricultural Center, 404 Life Sciences Building, Baton Rouge, LA 70803, USA; jeffdavis@agcenter.lsu.edu; 2 School of Animal Sciences, Louisiana State University Agricultural Center, 201D Animal & Food Sciences Building, Baton Rouge, LA 70803, USA; cli35@lsu.edu

**Keywords:** natural enemies, predator-prey interactions, stink bugs, soybean phenology

## Abstract

**Simple Summary:**

Soybean agroecosystems in the Midsouth support an assorted insect community. Louisiana soybean is heavily managed by growers, and the subsequent effect on residing arthropod populations is not well understood. For sustainable management of soybean, evaluations need not only focus on problematic pests, but also the resident natural enemies. This study addresses the compositional and temporal abundance of prominent insects in Louisiana soybean, both pest and beneficial. Pest and natural enemy populations were monitored to delineate potential associations. We found that the pest community was dominated by those that fed on soybean through piercing-sucking mouthparts. Predator populations were comprised mainly of spiders and big-eyed bugs. Pest and predator populations were similarly more abundant later in the soybean reproductive period. A moderate positive linear relationship was observed between pest and predator communities indicating a temporal correlation. Here we present essential information about the pest and natural enemy communities residing in Louisiana.

**Abstract:**

Over the past two decades, management practices within Louisiana soybean production have shifted. Successful application of an integrated pest management (IPM) strategy requires an understanding of how these changes have affected predator-pest dynamics. Surveys monitoring foliage-foraging arthropod populations in soybean took place across six locations within Louisiana over six years (2012–2014 and 2015–2018). Temporal associations of pest groups, defoliating and piercing-sucking, and predator groups relating to soybean phenology were observed. Additionally, soybean maturity groups (III, IV, and V) were also evaluated to delineate potential differences. Results indicated higher abundances of piercing-sucking pests compared to defoliating pests across both datasets (2012–2014 and 2015–2018). Pest groups were more abundant in later soybean reproductive periods, mainly attributed to *Chrysodeixis includens* and *Piezodorus guildinii*. Predator populations were mainly comprised of Araneae and Geocoridae throughout the survey periods. From 2015 to 2018, soybean growth had a significant effect on total predator abundance with more predators present at the pod-fill and soybean maturity stage. Correlations between total pest abundance and total predators exhibited a moderate positive linear relationship. Soybean maturity groups only influenced piercing-sucking pest abundance, with later maturing groups (IV and V) having higher numbers. Thus, control tools and tactics aimed at controlling late season pests should be modified to avoid reducing predator populations.

## 1. Introduction

Understanding the ecological framework of an agroecosystem is a central theme of integrated pest management (IPM) [1]. Recognition of compositional patterns amongst both pest and predator complexes is needed for successful implementation of an IPM plan. Understanding how predator-pest dynamics shape temporal abundance will allow for harmonious use of different control tactics [2]. Effective application of management tactics may avert occurrences of pest outbreaks and lead to more sustainable approaches within the agroecosystem [3,4,5,6].

The utilization of natural enemies as a control tactic is a fundamental part of any pest management plan. Unlike conservation biological control tactics, the use of resident natural enemies does not require the manipulation of an agroecosystem [7]. In fact, pest management strategies often neglect the exploitation of natural controls and their preexisting function within the ecosystem. This ecological service provided by resident natural enemies is estimated to be worth approximately USD 4.5 billion annually [8] across multiple cropping systems [9,10,11,12,13,14,15]. In agronomic crops, the assemblage of foliar-foraging predators in Midwestern soybean can maintain soybean aphid, *Aphis glycines* Matsumura, below economically damaging levels [16]. In vegetable systems, Funderburk et al. [17] observed significant suppression of *Frankinella* spp. by its natural enemy, *Orius insidiosus* (Say), in untreated field peppers; predator-prey ratios reached 1:40 for the most abundant thrips species, *F. occidentalis* (Pergande). However, incorporation of established predator communities into integrated pest management plans requires contemporary knowledge of predator-pest dynamics within the target system.

Louisiana soybean production systems have gone through major developments since the completion of two prior survey studies [18,19]. The soybean pest complex, particularly regarding stink bugs, has changed extensively [20]. *Piezodorus guildinii* (Westwood) has become the predominant stink bug species observed in soybean [20]. This stink bug is more tolerant to insecticides, up to eight-fold less susceptible to pyrethroids and organophosphates than *Nezara viridula* (L.), necessitating increased chemical applications for control [21]. The repetitive use of chemical controls can alter natural enemy communities [22,23,24,25,26]. Additionally, the use of insecticidal seed treatments has become more prevalent since the conduction of the two prior arthropod surveys in Louisiana soybean [27]. Recently, a meta-analysis found that neonicotinoid seed treatments negatively affected natural enemy abundances [28]. How these changes to the Louisiana soybean agroecosystem have affected the current corresponding arthropod community is unknown.

An additional change to Louisiana soybean production has been the shift to early production systems. For over the past 20 years, soybean growers have switched to planting early maturing varieties to avoid drought and late season pest pressure [29]. Although prior studies have observed differences between early production systems and conventional systems [18,19,30,31], distinctions between maturity groups (MGs) deemed early production (MG III–V) have not been fully assessed. Soybean MGs may influence abundances of pests or predators as MG determines the initiation period for reproductive growth [32]. How the use of early production systems over the past 20 years has affected the soybean arthropod community and whether the current soybean varieties adhere to similar predator-pest dynamics is unresolved.

To address this current lack of information, this study was conducted to provide a baseline for the pest and natural enemy community located in Louisiana soybean production systems prior to insecticide applications. In this study, foliar pest and predator compositions and abundances within three soybean MGs were assessed, while also addressing differences across phenological time over an average soybean season. Additionally, pest and natural enemy communities were evaluated for associations. Sampling took place over six years and across six different locations in Louisiana (2012–2014, five locations; 2015–2018, three locations). The research presented provides fundamental information regarding the resident natural enemies and the present pest populations.

## 2. Materials and Methods

### 2.1. Field Plots

Blocks of soybean (0.01–0.02 ha) corresponding to three MGs, III, IV, and V, were planted across Louisiana at five locations from 2012 to 2014 and at three locations from 2015 to 2018 (Figure S1) [33]. The three selected MGs are currently the most planted across the soybean acreage within Louisiana. At each site, soybeans were grown following LSU Agricultural Center (AgCenter) recommended agronomic practices and planting dates [34]. Soybeans were planted at eight seeds per 0.30 m on 0.76 m centers. Information regarding sampling site, variety, and planting date for each year is summarized in Table S1. No insecticide applications were employed throughout the experiment except initial seed treatments. Fungicides were applied at pod-fill (R5) to control *Cercospora* leaf blight following LSU AgCenter recommendations.

### 2.2. Sampling

Each MG block was separated into six plots that were sampled weekly from the initial reproductive stage (R1) to maturity (R8) (June to September) [35]. Samples consisted of one set of 25 sweeps within each plot with a standard (38 cm diam.) sweep net for a total of 150 sweeps per MG per week. Over an average yearly sampling period, 10–14 total samplings were collected. To avoid oversampling, alternate rows were swept between consecutive weeks. Collected samples were individually bagged and brought back to the laboratory for further identification and documentation of present pest and predatory groups. Observed arthropods were separated into three functional categories: defoliating herbivorous pests, piercing-sucking herbivorous pests, and predatory arthropods. Defoliating herbivores were comprised of the most common lepidopteran pests, while the piercing-sucking herbivore category was made up of the stink bug pest complex, plus threecornered alfalfa hopper, *Spissistilus festinus* (Say). Species within each of these functional groups were chosen as they are deemed to be of potential economic importance. Using taxonomic keys [36], predatory insects were identified to Family, while spiders were identified to Order.

Because not all planting sites were sampled consistently across all years, data were separated into two sets for analyses: 2012–2014 and 2015–2018. Sampling data from 2017 were omitted due to infrequent weekly sampling that year. Defoliating pest and predatory group numbers collected at the plot level throughout the study were low, so total numbers for each pest and predator group per six plots at each sampling date were used for analysis. To maintain consistency in analysis, piercing-sucking herbivore pests were also totaled per six plots. Counts were further combined into the following plant growth stage categories: R1-R2 (flowering), R3-R4 (pod development), R5-R6 (seed development), R7-R8 (soybean maturity). Weekly total counts were averaged within each growth stage category as growth stage length is not consistent across observed stages. The predator groups Pentatomidae and Chrysopidae were excluded from both dataset analyses due to consistently low numbers throughout the survey period (Table 1).

### 2.3. Statistical Analysis

Analyses of pest and predator abundance data were completed using a generalized linear mixed model approach (GLMM) with R version 3.6.0 [37]. Each functional group was analyzed separately within each dataset (six total analyses). To understand how arthropod group, MG, and phenological stage affected abundance, the glmmpql function found in the package, MASS [37,38], was used to fit a mixed-effect model for each dataset. Arthropod group, MG, and plant growth stage were used as fixed effects, while year and planting site were treated as random effects. Count data were log(x + 1) transformed for each dataset to meet the normality assumption. The lsmeans package [37,39] was used to further evaluate effects and interactions found to be significant (*p* < 0.05) with a Tukey-corrected least-square means for multiple comparisons approach. To evaluate the relationship between natural enemy and pest populations, Pearson’s correlation coefficients were obtained. The total predator abundance was assessed against the total pest abundances and for each functional pest group, defoliating herbivorous pests and piercing-sucking herbivorous pests. Data points report the sum of the specified arthropod group for each site, growth stage, year, and MG combination per 150 sweeps. Count data were log(x + 1) transformed to fit the normality assumption.

## 3. Results

A total of 1512 samples were analyzed from six planting sites (2012–2014, five locations; 2015–2018, three locations) over six sampled years. Lists of the collected arthropods placed into their functional pest or taxonomic predator groups, in addition to their proportions and total numbers, can be found in Table 1 and Table 2. Pest analyses focused only on species of economic importance. The defoliating herbivorous pest group was comprised of six species from the Order Lepidoptera, whereas the piercing-sucking herbivorous pest group had eight species from the Order Hemiptera, seven pentatomid species and one membracid species (Table 2). Total numbers collected for defoliating and piercing-sucking herbivore pests were 2764 and 21,941 specimens, respectively. Predator specimens were classified into nine taxonomic predator groups and totaled 16,166 individuals (Table 1).

### 3.1. Pest Composition and Phenology

**Figure 1:**
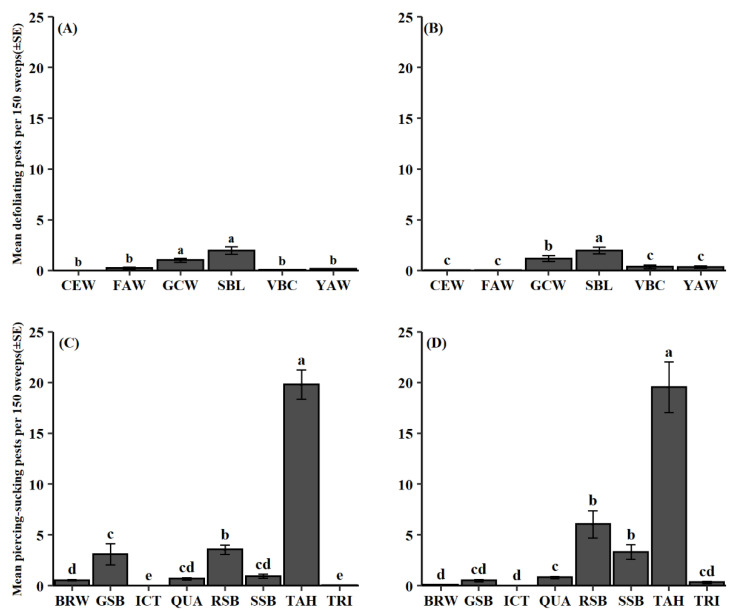
Mean pest count per 150 sweeps (±SE) for collected defoliating pest groups in datasets (**A**) 2012–2014 and (**B**) 2015–2018; piercing-sucking pest groups in datasets (**C**) 2012–2014 and (**D**) 2015–2018. Same letter denotes no significant difference between means (*p* > 0.05). Abbreviations for defoliating pest groups: CEW = *H. zea*, FAW = *S. frugiperda*, GCW = *H. scabra*, SBL = *C. includens*, VBC= *A. gemmatalis*, YAW = *S. ornithogalli*; piercing-sucking pests: BRW = *E. servus*, GSB = *C. hilaris*, ICT = *E. ictericus*, QUA = *E. quadrator*, RSB = *P. guildinii*, SSB = *N. viridula*, TAH = *S. festinus*, TRI = *E. tristigmus*.

Across both datasets, 2012–2014 and 2015–2018, and both pest categories, defoliating and piercing-sucking herbivorous pests, pest group had a significant effect (defoliating pests: 2012–2014, *F* = 49.07, df = 5, 984, *p* < 0.001; 2015–2018, *F* = 29.65, df = 5, 330, *p* < 0.001; piercing-sucking pests: 2012–2014, *F* = 387.60, df = 7, 1312, *p* < 0.001; 2015–2018, *F* = 173.33, df = 7, 440, *p* < 0.001). *Chrysodeixis includens* (Walker) was the most common defoliating pest followed by *Hypena scabra* (F.) in both the 2012–2014 and 2015–2018 datasets (Figure 1A,B). From 2012 to 2014 and 2015 to 2018, *S. festinus* Say was the most abundant piercing-sucking pest collected at 19.8 and 19.5 specimens per 150 sweeps, respectively (Figure 1C,D). Within the stink bug complex, *P. guildinii* was the most collected species in 2012–2014 and 2015–2018 at 3.5 and 6.0 specimens, respectively.

**Figure 2:**
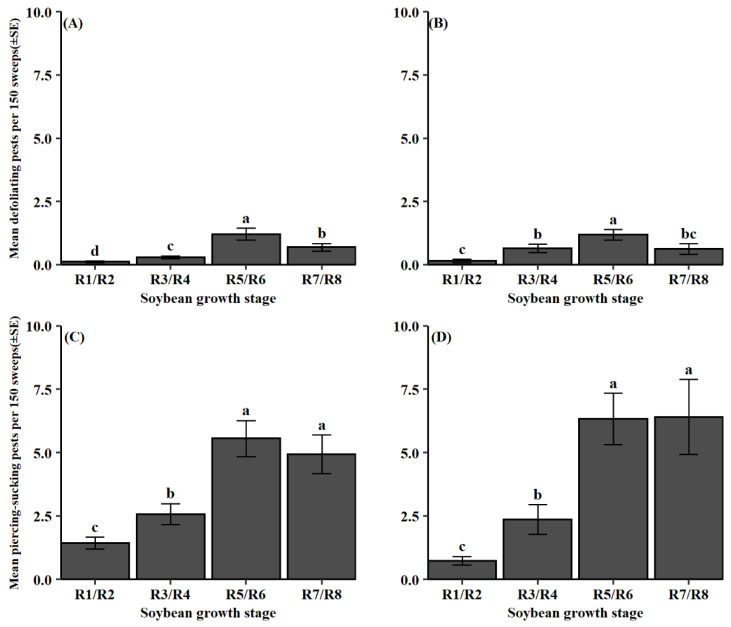
Mean pest count per 150 sweeps (±SE) for observed soybean growth stages of collected defoliating pest groups in datasets (**A**) 2012–2014 and (**B**) 2015–2018; piercing-sucking pest groups in datasets (**C**) 2012–2014 and (**D**) 2015–2018. Same letter denotes no significant difference between means (*p* > 0.05).

**Figure 3:**
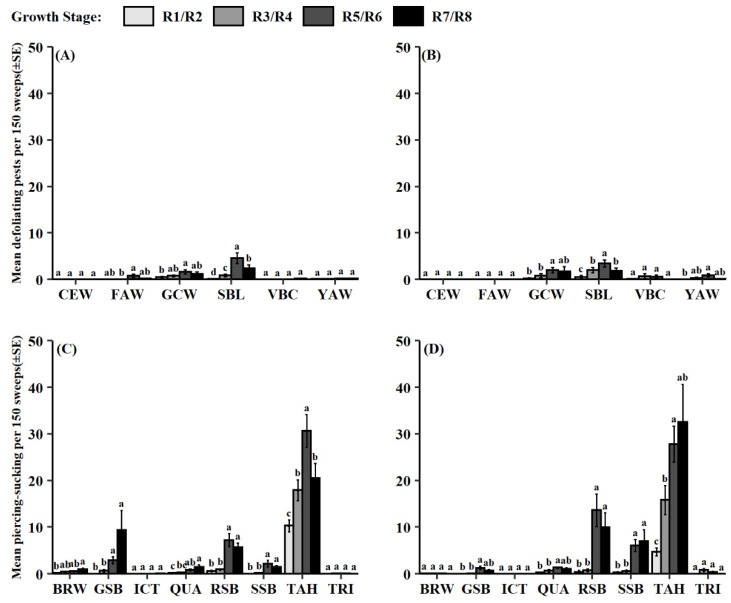
Mean pest count per 150 sweeps (±SE) for collected defoliating pest groups across soybean growth stages in datasets (**A**) 2012–2014 and (**B**) 2015–2018; piercing-sucking pest groups in datasets (**C**) 2012–2014 and (**D**) 2015–2018. Same letter denotes no significant difference between means (*p* > 0.05). Abbreviations for defoliating pest groups: CEW = *H. zea*, FAW = *S. frugiperda*, GCW = *H. scabra*, SBL = *C. includens*, VBC= *A. gemmatalis*, YAW = *S. ornithogalli*; piercing-sucking pests: BRW = *E. servus*, GSB = *C. hilaris*, ICT = *E. ictericus*, QUA = *E. quadrator*, RSB = *P. guildinii*, SSB = *N. viridula*, TAH = *S. festinus*, TRI = *E. tristigmus*.

Soybean growth stage had a significant effect on both functional pest groups during both dataset periods (defoliating pests: 2012–2014, *F* = 19.55, df = 3, 984, *p* < 0.001; 2015–2018, *F* = 15.53, df = 3, 330, *p* < 0.001; piercing-sucking pests: 2012–2014, *F* = 81.79, df = 3, 1312, *p* < 0.001; 2015–2018, *F* = 72.59, df = 3, 440, *p* < 0.001). In the 2012–2014 dataset, defoliating pests were most abundant during the pod-fill stage (R5/R6) followed by soybean maturity (R7/R8) (Figure 2A). From 2015 to 2018, defoliating pests were again most common during the pod-fill stage (R5/R6) (Figure 2B). Piercing-sucking pests were collected the most later in the soybean reproductive period during the pod-fill and soybean maturity stages (R5/R6 and R7/R8) in both datasets (Figure 2C,D). In addition, the interaction between pest group and soybean growth stage was significant for both functional pest groups and for each dataset (defoliating pests: 2012–2014, *F* = 6.63, df = 15, 984, *p* < 0.001; 2015–2018, *F* = 2.65, df = 15, 330, *p* < 0.001; piercing-sucking pests: 2012–2014, *F* = 8.86, df = 21, 1312, *p* < 0.001; 2015–2018, *F* = 11.12, df = 21, 440, *p* < 0.001). From 2012 to 2014, the defoliating species *C. includens*, *H. scabra*, and *Spodoptera frugiperda* (J.E. Smith) were most abundant during the pod-fill stage (R5/R6) (Figure 3A). Similar patterns were observed for 2015–2018 with *C. includens*, *H. scabra*, and *Spodoptera ornithogalli* (Guenée); all being collected more during the pod-fill stage (R5/R6) (Figure 3B). From the piercing-sucking pest category, *S. festinus* and *P. guildinii* were observed more frequently later in the reproductive period, pod-fill (R5/R6) and soybean maturity stages (R7/R8), in both datasets (Figure 3C,D). Less common piercing-sucking pest species followed similar patterns across both datasets. From 2012 to 2014, *Chinavia hilaris* (Say), *Euschistus quadrator* Rolston, *Euschistus servus* (Say), and *N. viridula* were found more during the pod-fill (R5/R6) and soybean maturity stage (R7/R8). From 2015 to 2018, *C. hilaris*, *E. quadrator*, and *N. viridula* had significantly higher numbers during the pod-fill (R5/R6) and soybean maturity stages (R7/R8).

For both datasets, soybean MG had a significant effect on piercing-sucking pests, but not defoliating pests (defoliating pests: 2012–2014, *F* = 1.72, df = 2, 984, *p* = 0.180; 2015–2018, *F* = 0.70, df = 2, 330, *p* = 0.496; piercing-sucking pests: 2012–2014, *F* = 6.45, df = 2, 1312, *p* < 0.002; 2015–2018, *F* = 5.04, df = 2, 440, *p* < 0.007). From 2012 to 2014, significantly more piercing-sucking pests were found in MG V compared to MG III, but MG V was not significantly more than MG IV (Figure S2A). From 2015 to 2018, MG IV had significantly more piercing-sucking pests collected compared to MG III, but not MG V (Figure S2B). No significant interaction between MG and pest group (defoliating pests: 2012–2014, *F* = 1.50, df = 10, 984, *p* = 0.133; 2015–2018, *F* = 0.85, df = 10, 330, *p* = 0.578; piercing-sucking pests: 2012–2014, *F* = 0.92, df = 14, 1312, *p* = 0.532; 2015–2018, *F* = 0.91, df = 14, 440, *p* = 0.550), nor MG and soybean growth stage (defoliating pests: 2012–2014, *F* = 0.58, df = 6, 984, *p* = 0.750; 2015–2018, *F* = 0.58, df = 6, 330, *p* = 0.743; piercing-sucking pests: 2012–2014, *F* = 1.03, df = 6, 1312, *p* = 0.404; 2015–2018, *F* = 1.57, df = 6, 440, *p* = 0.155) was observed for either functional pest group. Additionally, no three-way interaction between the fixed effects was observed (defoliating pests: 2012–2014, *F* = 0.44, df = 30, 984, *p* = 0.997; 2015–2018, *F* = 0.50, df = 30, 330, *p* = 0.989; piercing-sucking pests: 2012–2014, *F* = 0.47, df = 42, 1312, *p* = 0.999; 2015–2018, *F* = 0.64, df = 42, 440, *p* = 0.963).

### 3.2. Natural Enemy Composition and Phenology

**Figure 4:**
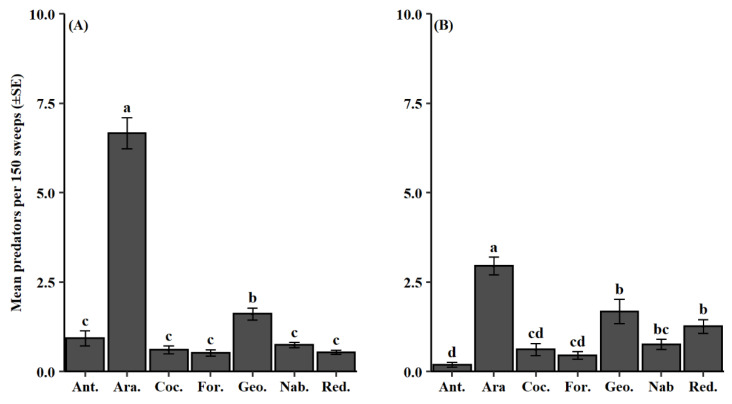
Mean predator count per 150 sweeps (±SE) for frequently collected predator groups in datasets (**A**) 2012–2014 and (**B**) 2015–2018. Same letter denotes no significant difference between means (*p* > 0.05). Abbreviations for predator groups are: Ant. = Anthocoridae, Coc. = Coccinellidae, For. = Formicidae, Geo. = Geocoridae, Nab. = Nabidae, and Red. = Reduviidae.

A significant predator group effect was observed in both the 2012–2014 and the 2015–2018 datasets (2012–2014, *F* = 163.08, df = 6, 1176, *p* < 0.001; 2015–2018, *F* = 40.231, df = 6, 581, *p* < 0.001). From 2012 to 2014, Araneae was collected significantly more than all other predator groups averaging 6.7 individuals per 150 sweeps over this period (Figure 4A). Geocoridae was the second most common group found during this sampling period with 1.6 individuals per 150 sweeps. All other predator groups were relatively low and showed no differences between their mean counts. Observations were similar from 2015 to 2018 with Araneae being the most dominant predator group with 3.0 individuals per 150 sweeps (Figure 4B). Geocoridae and Reduviidae had the next highest mean counts at 1.7 and 1.3 individuals per 150 sweeps, respectively.

**Figure 5:**
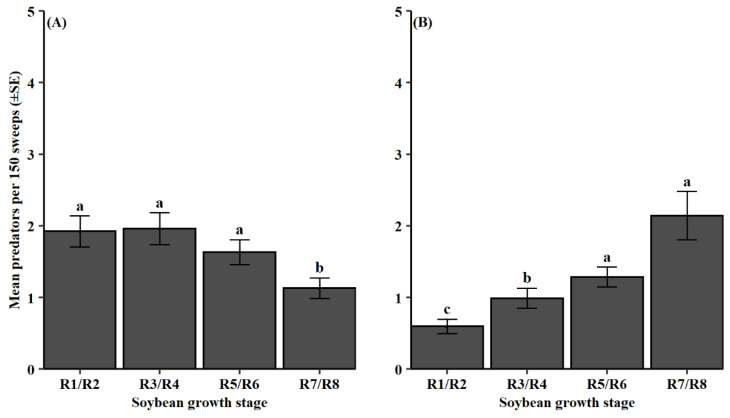
Mean predator count per 150 sweeps (±SE) for observed soybean growth stages during (**A**) 2012–2014 and (**B**) 2015–2018. Same letter denotes no significant difference between means (*p* > 0.05).

**Figure 6:**
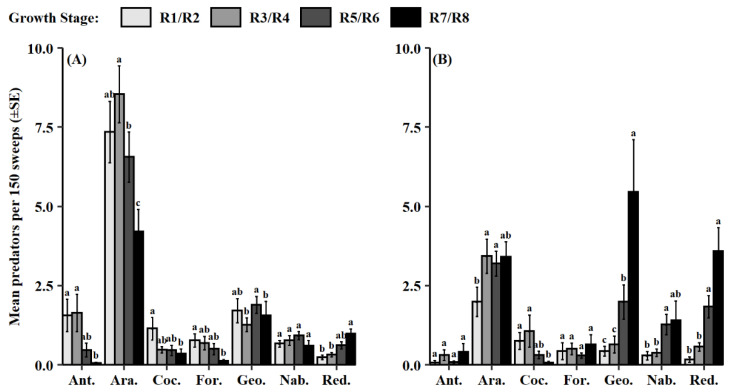
Mean predator count per 150 sweeps (±SE) for observed soybean growth stages across most common predator groups in datasets (**A**) 2012–2014 and (**B**) 2015–2018. Same letter denotes no significant difference within each predator group (*p* > 0.05). Abbreviations for predator groups are: Ant. = Anthocoridae, Coc. = Coccinellidae, For. = Formicidae, Geo. = Geocoridae, Nab. = Nabidae, and Red. = Reduviidae.

Soybean growth stage influenced predator counts in 2012–2014 and 2015–2018 (2012–2014, *F* = 8.47, df = 3, 1176, *p* < 0.001; 2015–2018, *F* = 19.04, df = 3, 581, *p* < 0.001). From 2012 to 2014, predators were significantly less abundant during soybean maturity (R7/R8) compared to the earlier growth stages (Figure 5A). For the 2015–2018 dataset, predator groups were collected significantly later in the soybean reproductive period during the pod-fill stage (R5/R6) and soybean maturity stage (R7/R8) (Figure 5B). Additionally, both datasets had significant predator group and growth stage interactions (2012–2014, *F* = 3.76, df = 18, 1176, *p* < 0.001; 2015–2018, *F* = 4.65, df = 18, 581, *p* < 0.001). From 2012 to 2014, the groups, Araneae and Anthocoridae, were most abundant during the pod-development stage (R3/R4) followed by the bloom stage (R1/R2) and then the pod-fill stage (R5/R6) (Figure 6A). Coccinellidae and Formicidae were observed earlier in the reproductive period during flowering (R1/R2) and pod-development stages (R3/R4). Geocoridae was most prominent at the pod-fill stage (R5/R6). In the latter dataset, Araneae was most frequently collected later in the reproductive period during the pod-development (R3/R4), pod-fill (R5/R6), and soybean maturity (R7/R8) stages (Figure 6B). Additionally, Geocoridae, Nabidae, and Reduviidae were most abundant at the soybean maturity stage (R7/R8).

MG did not have an effect on predator count in both datasets (2012–2014, *F* = 0.91, df = 2, 1176, *p* = 0.40; 2015–2018, *F* = 2.34, df = 2, 581, *p* = 0.10). The interaction between MG and predator group was not significant during both sampling periods (2012–2014, *F* = 0.30, df = 12, 1176, *p* = 0.99; 2015–2018, *F* = 0.40, df = 12, 581, *p* = 0.965). Conversely, the interaction between MG and plant growth stage had a significant effect on predator abundance from 2015 to 2018, but not within the 2012–2014 dataset (2012–2014, *F* = 0.99, df = 6, 1176, *p* = 0.431; 2015–2018, *F* = 2.13, df = 6, 581, *p* = 0.048). During the soybean maturity stage (R7/R8), significantly more predators were found in MG IV compared to MG V. There was no three-way interaction between predator group, MG, and growth stage for either period (2012–2014, *F*= 0.51, df = 24, 1176, *p* = 0.98; 2015–2018, *F* = 0.63, df = 24, 581, *p* = 0.91).

### 3.3. Pest and Natural Enemy Correlation

**Figure 7:**
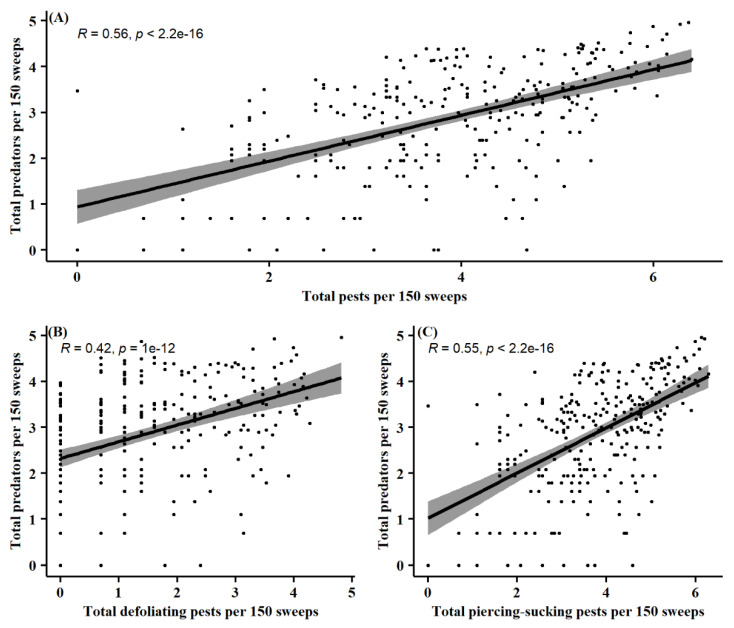
Pearson’s correlation coefficients comparing (**A**) per 150 sweeps, total predator abundance vs. total pest abundance, (**B**) total predator abundance vs. total defoliating pest abundance, and (**C**) total predator abundance vs. total piercing-sucking pest abundance. Data points represent each site, MG, growth stage, and year combination across the entire sampling period (2012–2018). Data point values are log(x + 1) transformations. *p* < 0.05 indicates significant correlation between variables.

Pearson’s correlation coefficients were determined to verify linear correlation between the pest and natural enemy community. Total pest abundance, as well as each individual functional pest group, was compared to total predator abundance per 150 sweeps for an overall of three separate correlations. A significant moderate positive linear correlation was found between total pest abundance and total predator abundance (*R* = 0.56, *p* < 0.001) (Figure 7A). When comparing total defoliating pest numbers to total predator numbers, a significant moderate positive linear correlation was observed (*R* = 0.42, *p* < 0.001) (Figure 7B). Additionally, total piercing-sucking pest abundances were shown to have a significant moderate positive linear correlation to total predator abundances (*R* = 0.55, *p* < 0.001) (Figure 7C).

## 4. Discussion

This study is the first to examine both the pest and predator composition in Louisiana soybean across a substantial time (six years). Observations over this period have allowed for recognition of compositional and phenological patterns within the soybean arthropod community. Here we present information and insights that will guide soybean growers in the development of more sustainable management decisions.

Throughout this study, observed defoliator numbers were relatively low in comparison to their piercing-sucking counterparts (Figure 1). This divergence is the result of early soybean production system adoption, which has allowed farmers to temporally escape lepidopteran pests that migrate to Louisiana soybean later in the growing season [29]. In response to this shift, *C. includens* has become the dominant lepidopteran pest, while the pest previously regarded as the most economically important, *Anticarsia gemmatalis* Hübner [40], has become absent (Figure 1A,B). Although *C. includens* abundances were low throughout this study, resistance to multiple broad-spectrum insecticide classes [41] may increase potential risk of a secondary pest outbreak of this species [42]. Further evaluation of current insecticidal controls and their potential to induce outbreak is needed.

Previous studies have shown that with a shift to early soybean production systems comes an increasing risk for economically damaging population of piercing-sucking insect pests [19,31,43,44,45]. In this study, *S. festinus* was the most dominant piercing-sucking herbivorous pest, which agrees with previous results showing larger abundances in early production systems (Figure 1C,D) [19,45]. The stink bug complex reached its action threshold (16 stink bugs per 100 sweeps) in about 60% of the sampling site, MG, and year combinations. *P. guildinii* was the most abundantly collected stink bug over the duration of this study followed by either *C. hilaris* (2012–2014) or *N. viridula* (2015–2018) (Figure 1C,D). Since its establishment in the early 2000s [46], *P. guildinii* has become the dominant stink bug pest [20]. Reduced susceptibility to broad-spectrum controls [21], in addition to its adaption to subtropical climates [47,48,49], have allowed *P. guildinii* to prosper in Louisiana soybean. Current means of control for *P. guildinii* focus strictly on chemical controls. Further study is needed for development of alternative and more sustainable approaches to control this pest.

A similar compositional pattern in predator abundances was observed between the 2012–2014 and 2015–2018 dataset. Araneae was the most abundant arthropod group collected averaging 6.7 and 3.0 individuals per 150 sweeps in 2012–2014 and 2015–2018, respectively (Figure 4). Geocoridae was the second most common predator group averaging 1.6 and 1.7 individuals per 150 sweeps in 2012–2014 and 2015–2018, respectively. These results are consistent with previous studies surveying arthropod predators in Louisiana soybean, which found Araneae and Geocoridae to be the most abundant [18,19]. These two groups have remained dominant for almost 20 years despite the non-persistent nature of Louisiana soybean agroecosystems. Now, whether these groups provide a value through natural pest control remains to be described. Past studies have presented inconsistent findings regarding the importance of groups like Geocoridae and the impact they have on soybean pests [50,51]. Additionally, Araneae has exhibited behaviors of intraguild predation in soybean [52,53], which is counterproductive to their desired effect. Additional information is needed to elucidate the effect common predators like Araneae and Geocoridae have on prominent pests in Louisiana soybean.

Understanding when pest and predator populations are present during a growing season is important to optimally apply IPM tactics. Results from this study agree with prior reports, which indicate larger abundances of lepidopteran and stink bug groups later in the soybean reproductive period [20,54]. Defoliating species reduced once soybean maturity (R7/R8) was reached, while piercing-sucking species populations sustained (Figure 2). This disparity may relate to the increased fiber composition of mature soybean leaves [55], prompting movement to younger plants. Due to their feeding method and preference, piercing-sucking pests like stink bugs are predicted to not be affected by this change [56]. Overall, growth stage patterns were driven by the most abundant pest species found within each functional pest group (Figure 1), as these species had similar phenological patterns (Figure 3).

Conversely, predator populations were temporally more variable between the two datasets (Figure 5). Abundances of predators were reduced by the time of soybean maturity (R7/R8) in 2012–2014 but continued to grow into this phenological stage from 2015 to 2018. This difference was in part due to the observed hemipteran predators, Geocoridae, Nabidae, and Reduviidae, which were all present at higher numbers in more mature soybean (R7/R8) during the 2015–2018 dataset (Figure 6B). This agrees with the prior observations from Shepard et al. [57], which also saw predator population peaks later in the soybean growing season. Causation of these phenological patterns may be related to increased pest populations (Figure 7). Derived correlation coefficients showed moderate positive linear relationships between total predator abundance and total pest abundance, as well as total predator abundance and each categorized functional pest group. A positive numerical response to increased prey density is characteristic of natural enemies [58]. Araneae, Geocoride, and Nabidae have all been shown prior to be positively correlated with lepidopteran larval populations in soybean [30]. This raises some concern as insecticide application and peak predator density may concurrently occur. Consequently, predators are at an increased risk of exposure to these chemical controls.

Integration of the endemic natural enemies into Louisiana soybean IPM strategies will require compatibility with chemical controls. Application of insecticides is needed when predators are incapable of keeping pest populations in check as seen with stink bug populations within this experimental period. Insecticides are a common control tool used for pests in Louisiana soybean, averaging three applications over a single season [59]. Non-target effects of insecticides on natural enemies in soybean have been thoroughly examined [60,61,62,63,64,65,66,67,68]. Both Araneae and Geocoridae, the most prominent groups in this study, are susceptible to broad spectrum insecticides like pyrethroids and organophosphates. Additionally, Geocoridae is a facultative plant feeder in addition to insect prey [69]. Plant feeding exposes Geocoridae to systemic insecticides like neonicotinoids, which have been shown to be toxic to this group [70]. Imidacloprid and thiamethoxam both caused 100% mortality of *Geocoris punctipes* (Say) ten days after feeding on systemically treated leaves. More selective insecticides will be necessary to avoid reduction in natural enemy abundance and subsequently the potential for secondary pest outbreak. Further studies will be needed to assess how these insecticides impact natural enemy abundances.

The use of conservation biological control tactics may be sought to enhance the soybean natural enemy community. Conservation biological control practices have the potential to increase natural enemy effectiveness through provision of additional food sources or refugia [71,72,73,74]. Araneae was 88% more abundant in plots of spring wheat mulched with barley straw compared to those where mulch was absent [75]. Similarly, cucumber interplanted with living red clover mulch produced significant increases in Geocoridae abundances [76]. Although some tactics may provide advantages, realization of these benefits may be context or farm dependent [77]. Moore et al. [78] found that floral resource producing partridge pea, *Chamaecrista fasciculata* (Michx.) Greene, surprisingly acted as a sink for natural enemies, leading them away from adjacent soybean. Verification is needed to address whether possible conservation tactics can be applied to increase predator effectiveness in Louisiana soybean and if said practices can be incorporated into current management plans.

Early soybean production systems are used exclusively by Louisiana soybean growers [20], but dissimilarities in present arthropod communities have not yet been established between the accepted “early” maturing groups. Boyd et al. [18] and McPherson et al. [45] did survey different early maturing groups, but no statistical comparison was made nor was MG III included. Within the two datasets of this study, MG was found to have an effect only on the piercing-sucking herbivorous pests. Piercing-sucking herbivorous pests were more abundant in MG IV and V compared to MG III. Group IIIs mature faster than the other observed groups [79], which may have an effect on piercing-sucking pests like stink bugs that generally feed on the soybean pod [56]. While there was no main effect of MG on predator populations throughout this study, there was an interaction between MG and soybean growth stage. Predators collected during the soybean maturity stage (R7/R8) were more abundant in MG III and IV. Reasons for this finding are unclear as observed functional pest groups showed a different pattern of abundance between the experimental MGs used. Previous reports on the effects of MG on natural enemy abundance have been inconsistent [18,19,31]. Pest groups outside the scope of this study may have a role in the observed pattern and further investigation will be required. Overall, the minimal effect of MG on endemic predators will make the combination of early maturing varieties and biological tactics viable for Louisiana soybean growers.

## 5. Conclusions

In this study, predator-pest dynamics of arthropod communities in Louisiana soybean were addressed. The switch to early soybean production by Louisiana growers has shifted the pest complex, resulting in higher abundances of piercing-sucking herbivorous pests. Although the endemic natural enemy community was correlated to observed pest populations, economic threshold levels were still reached by piercing-sucking herbivorous pests. Early season establishment of predators may limit early “explosion” of pest populations [80,81]. Assessment of conservation biological control tactics and their capacity to foster the establishment of natural enemies in Louisiana soybean is needed. Successful integration of natural enemies into an IPM program will increase sustainability through herbivore suppression and reduced insecticide use. When natural enemies cannot contain pests below economic thresholds, the use of insecticides is expected. Certain soybean pests like *P. guildinii* are less susceptible to current broad-spectrum controls and may require multiple applications [21]. Further study is required to evaluate more selective controls for stink bug pests in Louisiana soybean that do not harm natural enemies. Tandem use of chemical and biological controls may reduce the occurrence of resurgence or secondary pest outbreaks and is a cornerstone of IPM.

## Figures and Tables

**Table 1:** Relative abundance and total count data for collected predator groups, 2012–2018.

Classification		Relative Abundance (Total Count)		
Order	Family (Abbr.)	2012–2014	2015–2018	Total
Coleoptera	Coccinellidae (Coc.)	4.8 (267)	6.7 (164)	5.4 (431)
Hemiptera	Anthocoridae (Ant.)	7.6 (426)	2.1 (51)	5.9 (477)
Hemiptera	Geocoridae (Geo.)	14.7 (827)	18.1 (441)	15.8 (1268)
Hemiptera	Nabidae (Nab.)	7.1 (400)	10.4 (252)	8.1 (652)
Hemiptera	Pentatomidae	0.9 (48)	1.2 (30)	1.0 (78)
Hemiptera	Reduviidae (Red.)	4.9 (273)	16.1 (391)	8.3 (664)
Hymenoptera	Formicidae (For.)	4.0 (225)	4.4 (107)	4.1 (332)
Neuroptera	Chrysopidae	0.8 (42)	1.4 (33)	0.9 (75)
Araneae	NA (Ara.)	55.3 (3104)	39.7 (965)	50.6 (4069)

**Table 2:** Relative abundance and total count data for collected pest groups, defoliating and piercing-sucking herbivores, 2012–2018.

Defoliating Herbivorous Pests				
Classification		Relative Abundance (Total Count)		
Species	Common Name (Abbr.)	2012–2014	2015–2018	Total
*Anticarsia gemmatalis*	Velvetbean caterpillar (VBC)	1.3 (22)	7.9 (83)	3.8 (105)
*Chrysodeixis includens*	Soybean looper (SBL)	49.7 (853)	50.3 (528)	50.0 (1381)
*Helicoverpa zea*	Corn earworm (CEW)	0.2 (3)	0.5 (5)	0.3 (8)
*Hypena scabra*	Green cloverworm (GCW)	32.7 (560)	31.7 (333)	32.3 (893)
*Spodoptera frugiperda*	Fall armyworm (FAW)	11.8 (203)	1.9 (20)	8.1 (223)
*Spodoptera ornithogalli*	Yellowstriped armyworm (YAW)	4.3 (74)	7.6 (80)	5.6 (154)
**Piercing-Sucking Herbivorous Pests**				
**Classification**		**Relative Abundance (Total Count)**		
Species	Common Name (Abbr.)	2012–2014	2015–2018	Total
*Chinavia hilaris*	Green stink bug (GSB)	10.0 (1383)	2.0 (158)	7.0 (273)
*Euschistus ictericus*	NA (ICT)	0.1 (10)	>0.1 (2)	0.1 (12)
*Euschistus quadrator*	NA (QUA)	2.5 (344)	2.7 (221)	2.6 (565)
*Euschistus servus*	Brown stink bug (BRW)	1.8 (248)	0.3 (25)	1.2 (273)
*Euschistus tristigmus*	Dusky stink bug (TRI)	0.3 (37)	0.9 (75)	0.5 (112)
*Nezara viridula*	Southern green stink bug (SSB)	4.2 (586)	10.2 (824)	6.4 (1410)
*Piezodorus guildinii*	Redbanded stink bug (RSB)	12.5 (1734)	21.8 (1758)	15.9 (3484)
*Spissistilus festinus*	Threecornered alfalfa hopper (TAH)	68.7 (9531)	62.1 (5013)	66.3 (14544)

## Data Availability

There are no publicly accessible archives of the data.

## Supplementary Materials

The following supporting information can be downloaded at: https://www.mdpi.com/article/10.3390/insects13020154/s1, Figure S1: Sampling sites within Louisiana; Figure S2: Comparison of piercing-sucking pest abundances amongst observed MGs; Table S1: Sampling locations, MGs, soybean varieties, and planting dates, 2012–2018.
