# Supplementary material for: Predator-Pest Dynamics of Arthropods Residing in Louisiana Soybean Agroecosystems
Source: Insects. 2022 Jan 31;13(2):154. doi: 10.3390/insects13020154 (PMC8875837; doi:10.3390/insects13020154)
Supplement: Supplementary file 1 [file insects-13-00154-s001.zip › insects-1573346-supplementary/SLee_Pred_Pest_2022_Supplementary.pdf]

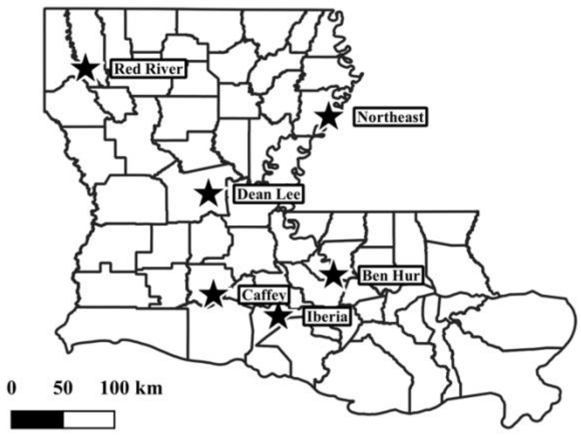

Figure S1. Sampling site locations of pest and predator surveys across Louisiana parishes [33].

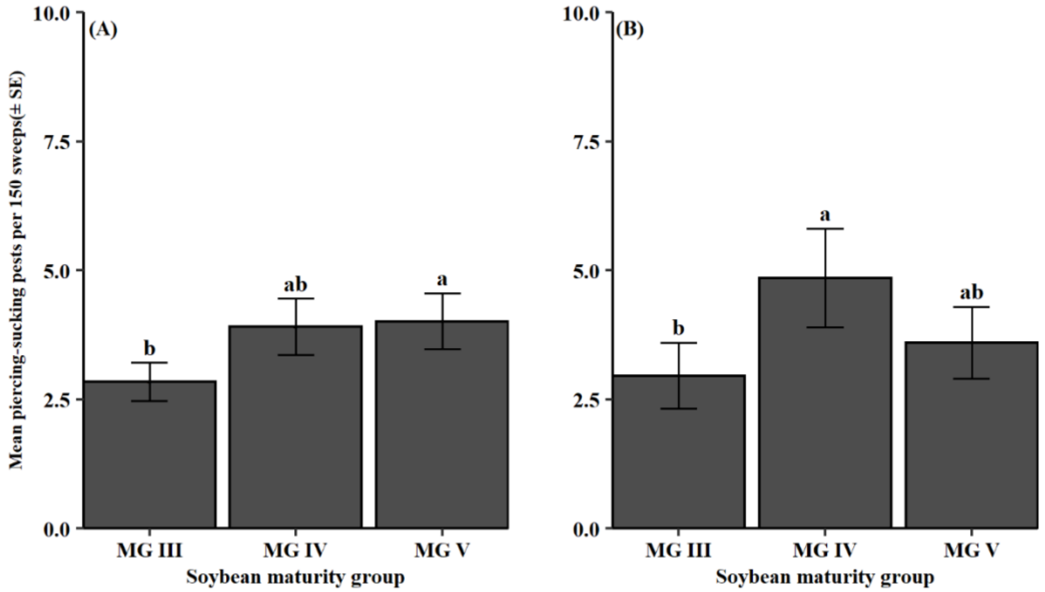

Figure S2. Mean pest count per 150 sweeps (±SE) for observed maturity groups (MG) of collected sucking pest groups in datasets (**A**) 2012–2014 and (**B**) 2015–2018. Same letter denotes no significant difference between means ($p > 0.05$).

Table S1. Sampling location, maturity group (MG), soybean variety, and planting date for Louisiana predator survey, 2012-2018.

Planting Site (Station Coordinates)	MG	Soybean Variety (Planting date)					
		2012	2013	2014	2015	2016	2018
Ben Hur Research Station (30°22'7.79"N, 91° 9'59.57"W)	III	Pioneer 93Y92 (May 7)	Pioneer 93Y92 (May 17)	Pioneer 93Y92 (April 30)	Asgrow 4034 (May 8)	Pioneer 93Y92 (May 5)	Asgrow 40X6 (May 30)
	IV	Asgrow 4605 (May 7)	Asgrow 4605 (May 17)	Asgrow 4605 (April 30)	Asgrow 4633 (May 8)	Asgrow 4533 (May 5)	Asgrow 46X6 (May 30)
	V	Pioneer 95Y20 (May 7)	Asgrow 5605 (May 17)	Asgrow 5605 (April 30)	Asgrow 5535 (May 8)	Asgrow 5633 (May 5)	Asgrow 56X8 (May 30)
Caffey Research Station- S. Farm (30°10'32.22"N, 92°21'41.81"W)	III	Pioneer 93Y92 (May 15)	Pioneer 93Y92 (May 10)	Pioneer 93Y92 (April 28)	NA	NA	NA
	IV	Asgrow 4605 (May 15)	Asgrow 4605 (May 10)	Asgrow 4605 (April 28)	NA	NA	NA
	V	Asgrow 5605 (May 15)	Asgrow 5605 (May 10)	Asgrow 5605 (April 28)	NA	NA	NA
Dean Lee Research Station (31°10'40.28"N, 92°24'37.69"W)	III	Pioneer 93Y92 (April 27)	Pioneer 93Y92 (May 15)	Pioneer 93Y92 (May 1)	LibertyCZ3945LL (May 15)	Pioneer 93Y92 (May 24)	Asgrow 40X6 (May 24)
	IV	Asgrow 4605 (April 27)	Asgrow 4605 (May 15)	Asgrow 4605 (May 1)	LibertyCZ4748LL (May 15)	Asgrow 4533 (May 24)	Asgrow 46X6 (May 24)
	V	Asgrow 5605 (April 27)	Asgrow 5605 (May 15)	Asgrow 5605 (May 1)	LibertyCZ5445LL (May 15)	Asgrow 5633 (May 24)	Asgrow 56X8 (May 24)
Iberia Research Station (29°57'41.26"N, 91°42'58.96"W)	III	NA	NA	NA	Asgrow 4034 (April 23)	Pioneer 93Y92 (April 28)	Asgrow 40X6 (April 24)
	IV	NA	NA	NA	Asgrow 4633 (April 23)	Asgrow 4533 (April 28)	Asgrow 46X6 (April 24)
	V	NA	NA	NA	Asgrow 5535 (April 23)	Asgrow 5633 (April 28)	Asgrow 56X8 (April 24)
Red River Research Station (32°25'20.79"N, 93°38'4.40"W)	III	Pioneer 93Y92 (May 7)	Pioneer 93Y92 (April 29)	Pioneer 93Y92 (May 16)	NA	NA	NA
	IV	Asgrow 4605 (May 7)	Asgrow 4605 (April 29)	Asgrow 4605 (May 16)	NA	NA	NA
	V	Asgrow 5605 (May 7)	Asgrow 5605 (April 29)	Asgrow 5605 (May 16)	NA	NA	NA
Northeast Research Station (31°56'37.72"N, 91°12'54.98"W)	III	Pioneer 93Y92 (May 3)	Pioneer 93Y92 (April 22)	Pioneer 93Y92 (May 14)	NA	NA	NA
	IV	Asgrow 4605 (May 3)	Asgrow 4605 (April 22)	Asgrow 4605 (May 14)	NA	NA	NA
	V	Asgrow 5905 (May 3)	Asgrow 5605 (April 22)	Asgrow 5605 (May 14)	NA	NA	NA
